# Photoacoustic stimulation promotes the osteogenic differentiation of bone mesenchymal stem cells to enhance the repair of bone defect

**DOI:** 10.1038/s41598-017-15879-4

**Published:** 2017-11-20

**Authors:** Zebin Huang, Jiankun Xu, Jiebin Chen, Hongjiang Chen, Hailong Wang, Zhonglian Huang, Youbin Chen, Xiaolin Lu, Fushen Lu, Jun Hu

**Affiliations:** 1 Department of Orthopaedics, the First Affiliated Hospital, Shantou University Medical College, Guangdong Province, China; 2 Department of Chemistry and Key Laboratory for Preparation and Application of Ordered Structural Materials of Guangdong Province, Shantou University, Guangdong Province, China; 3 Department of Orthopaedics and Traumatology, Prince of Wales Hospital, Faculty of Medicine, the Chinese University of Hong Kong, Hong Kong SAR, China

## Abstract

The aim of this study was to evaluate the direct photoacoustic (PA) effect on bone marrow mesenchymal stem cells (BMSCs) which is a key cell source for osteogenesis. As scaffold is also an indispensable element for tissue regeneration, here we firstly fabricated a composited sheet using polylactic-co-glycolic acid (PLGA) mixing with graphene oxide (GO). BMSCs were seeded on the PLGA-GO sheets and received PA treatment *in vitro* for 3, 9 and 15 days, respectively. Then the BMSCs were harvested and subjected to assess alkaline phosphatase (ALP) activity, calcium content and osteopontin (OPN) on 3, 9 and 15 days. For *in vivo* study, PLGA-GO sheet seeded with BMSCs after *in vitro* PA stimulation for 9 days were implanted to repair the bone defect established in the femoral mid-shaft of Sprague-Dawley rat. PLGA-GO group with PA pretreatment showed promising outcomes in terms of the expression of ALP, OPN, and calcium content, thus enhanced the repair of bone defect. In conclusion, we have developed an alternative approach to enhance the repair of bone defect by making good use of the beneficial effect of PA.

## Introduction

Bone defects and fracture delay union or non-union are commonly refractory diseases caused by arthritis, trauma or tumor excision. Currently, the most effective treatment is the utilization of bone autografts and bone allografts. More than 2.2 million bone grafts have been used in clinical application every year worldwide^1^. However, apart from the limited supply, bone grafts may cause some other potential problems, such as disease transfer, histo-incompatibilities, re-fracture and secondary infection^1–3^. Hence, how to efficiently repair bone defect remains a great challenge. It is highly desirable to enhance the local blood supply and further support bone formation to fill the gap at the bone defect site. Involuntarily, looking for more effective treatment to enhance both osteogenesis and angiogenesis while without the limitations and drawbacks of traditional therapies has become a hot topic of concern.

With the development of bone tissue engineering, composite biomaterials with good biocompatibility as a carrier providing sufficient growth space to not only support the proliferation and differentiation of the cells, but also direct 3D tissue formation are widely used^4–6^. *Hemin Nie et al*. succeed in applying polylactic-co-glycolic acid (PLGA) scaffold to deliver BMP-2 for enhancing bone defect repair^7^. Recently, graphene is characterized as a biomimetic nanomaterial with special physical properties and structures and thus is proposed for numerous biomedical applications^8^. Graphene oxide (GO), as the derivatives of graphene, has a better performance in terms of structure and biological properties as compared to graphene. GO (either functionalized one or released one from polymeric composites) is of excellent bio-compatibility and can be eliminated through renal and fecal routes^9–11^. Furthermore, appropriate ratio of GO is able to improve both the thermomechanical properties and biocompatibility of composite biomaterial properties^12^. Moreover, both forms are able to induce the BMSCs differentiate into osteoblasts^8,12–14^. *Xiaoming Li et al*. have successfully induced the human bone mesenchymal stem cells osteogenesis and ectopic ossification by using the single-walled carbon nanotubes^15^. Additionally, of note, the photoacoustic (PA) effect, media volume occur periodically harmomegathus due to internal temperature change producing physical acoustic wave when the pulse light source or modulation light source stimulate on the medium^16^, have been used widely in imaging and spectroscopy in material sciences, engineering and medicine since its discovery in 1881^17–19^. Recently, several studies consistently reveal that PA effect has promising capacity to promote BMSCs differentiate into osteoblasts^17,20^.

Currently, in the field of investigation on the treatment of bone defect, the overall concept mainly focuses on the composited materials combined with bioactive molecules or nanomaterials^21,22^. However, the effect of PA on the cells seeding on the composite scaffolds to repair the bone defect is largely unknown. In our study, a PLGA biological composite scaffold mixing with GO were designed and fabricated as a carrier for the BMSCs, combining with PA effect to develop an alternative and innovative approach to effectively repair the bone defect. PLGA was chosen as basic skeleton of porous scaffold for its biocompatibility and malleability^23^, and adding appropriate ratio of GO is not only able to amplify the PA effect after being stimulated with pulsed laser, but also to enhance both thermomechanical properties and biocompatibility of PLGA scaffold^24^. As the BMSCs are of multi-lineage differentiation potentials that can differentiate into osteoblasts, chondrocytes, adipocytes, and neurocytes under properly induction^25,26^, they have been widely used in the bone tissue-engineering. Therefore, we used the PLGA-GO seeding with BMSCs in alliance with PA effect as an organic unit for promoting BMSCs osteogenic differentiation *in vitro* and repairing the bone defect model established in a Sprague-Dawley rat *in vivo*. In our *in vitro* experiments, the BMSCs was cultivated in dish with GO, after treating with pulsed laser, the osteogenetic differentiation potential of the BMSCs have be investigated. For *in vivo* experiments, the PLGA-GO scaffold, seeded with BMSCs and stimulated *in vitro* with pulsed laser, was implanted into the bone defect site created on the mid-shaft of femur in rat. We have also adopted X-ray, micro-computed tomography (micro-CT), immunohistochemistry to comprehensively evaluate the therapeutic effect of this newly developed organic unit in the repair of bone defect.

## Results

### PLGA/GO scaffolds were successfully fabricated and characterized

**Figure 1:**
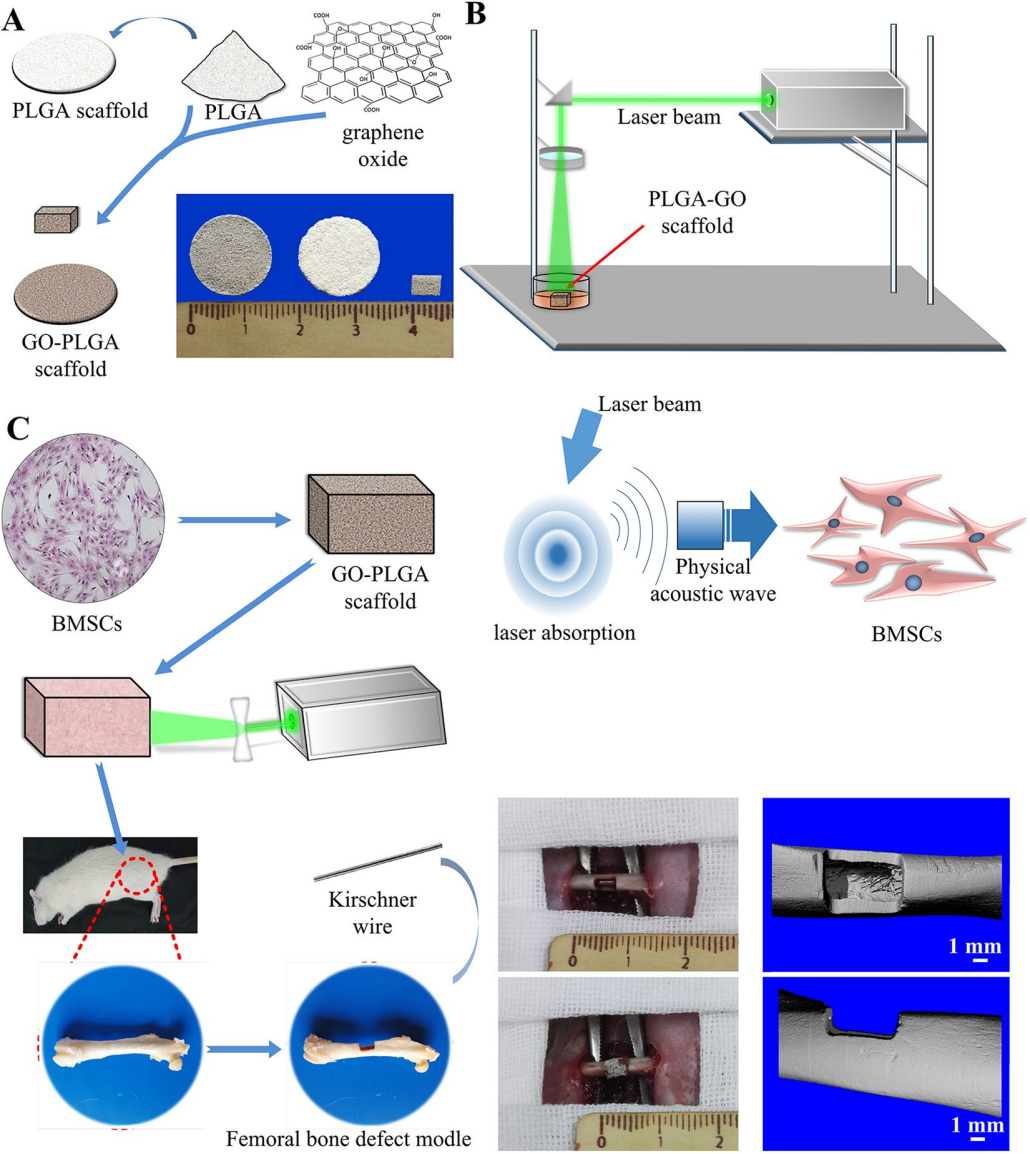
(**A**) Schematic diagram showing the process for manufacturing the PLGA-GO scaffold. (**B**) Schematic diagram showing the setup of PA treatment platform. (**C**) The images showing the establishment of bone defect model at the femoral mid-shaft of rat.

**Figure 2:**
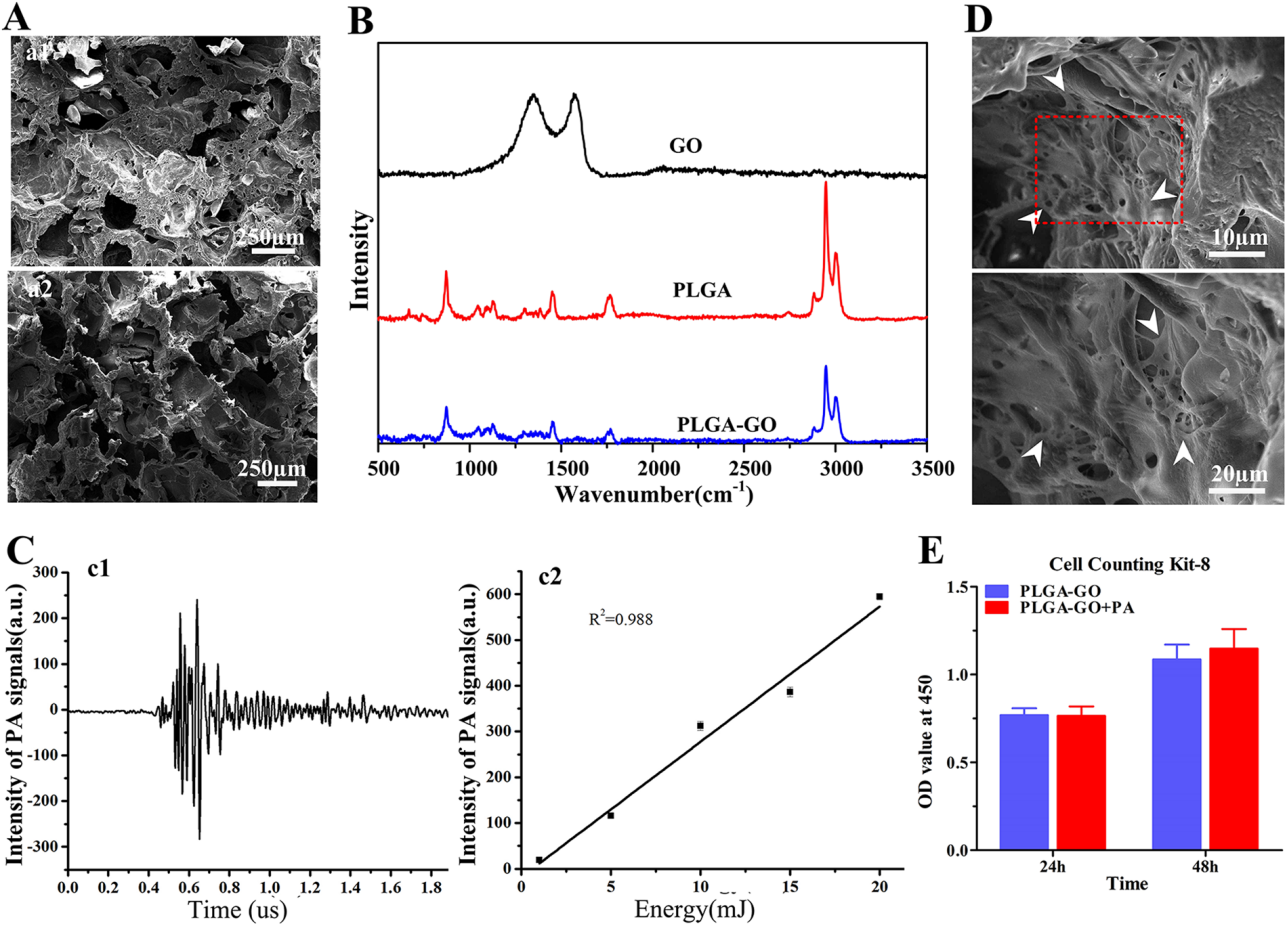
Characterization of the fabricated scaffolds *in vitro*. (**A**) The microstructures of PLGA-GO scaffolds (a1) and PLGA scaffolds (a2) under the scanning electron microscope. (**B**) Raman spectra of GO, pristine PLGA film and PLGA-GO film. (**C**) Under the 10 mJ pulsed laser stimulating, graph (c1) showed the PA signal producing by PLGA-GO scaffolds; graph (c2) showed the PA signal of PLGA-GO scaffolds with different intensities of Laser. (**D**) Scanning electron microscopy images showed that BMSCs attached and spread well on the surface of PLGA-GO scaffold after cultivating 72 h. White arrows indicated the BMSCs. (**E**) Proliferation tests of BMSCs growing on PLGA-GO scaffolds with or with PA stimulation at 24 h and 48 h. All quantitative data were presented as mean ± SD, n = 4. No statistical difference was found between group as indicated by unpaired two-tailed Student’s *t* test.

To fabricate porous PLGA/GO scaffolds with appropriate pore sizes for the growth and proliferation of BMSCs (Fig. 1A), NaCl particles (size: 200-300 μm) were used as porogen. As shown in scanning electron microscopy (SEM) images (Fig. 2A), both PLGA and PLGA-GO scaffolds had a porous structure. According to the True Density Analyzer (TD-2200), the porosity of PLGA and PLGA-GO scaffolds were calculated to be 88.7% and 87.5%, respectively. Theoretically, both the porosity and pore size of PLGA and PLGA-GO should satisfy the growth and reproduction of BMSCs^27^.

GO exhibited two peaks at 1348 cm^−1^ and 1580 cm^−1^, corresponding to the D band of defective structure and the G band of graphitic structure, respectively^28,29^, PLGA film exhibited two bands at 874 cm^−1^ and 1764 cm^−1^, corresponding to stretching of –C-COO and C=O, respectively^30,31^. The peaks at 1452 cm^−1^and 2900 cm^−1^ were attributed to the bending and stretching vibration of –C-H bonds in the backbone and side chains of PLGA, respectively (Fig. 2B). PLGA-GO showed the same peaks as PLGA did (Fig. 2B). However, the D and G bands of GO were not detectable in the PLGA-GO Raman spectrum, which are likely due to the rough weak signal of tiny amount or homogenous dispersion of GO in PLGA-GO composites.

In terms of laser energy, we could detect the photoacoustic signal of PLGA-GO scaffold when it was stimulated with pulsed laser with an energy of 10 mJ (Fig. 2C,c1). Under diverse intensity of laser beam, PLGA-GO scaffold exposure laser beam produces significant PA signal showing a positive correlation to laser energy (Fig. 2C,c2).

### The ratio of GO was optimized as 0.16%

To reduce the cytotoxicity of GO to primary cultured BMSCs (Supplementary Figs 1, 2), we firstly optimized the ratio of GO in the PLGA scaffold (Supplementary Fig. 4). No more than 0.16% (in weight) of GO in PLGA showed no inhibition on cell growth (Supplementary Fig. 4). In addition, the incorporation of GO (up to 1 wt%) into PLGA did not obviously alter the degradation of PLGA, which had been revealed by the kinetics studies and SEM images of GO-PLGA composites, consisting with the literature^32,33^. Then we also seeded BMSCs on the scaffold and observed by SEM after 72 h cultivating. The electron microscope image demonstrates that BMSCs can grow and spread well on the PLGA-GO scaffolds, even covering the surface (Fig. 2D).

### The energy of PA was optimized as 10 mJ

Per the cell proliferation test, the optimal energy of pulsed laser was set as 10 mJ, which showed no deleterious effect on cell growth (Supplementary Fig. 3). In addition, when the BMSCs were seeded on PLGA-GO scaffold, 10 mJ PA treatment did not affect cell proliferation, as compared to those without PA treatment (Fig. 2E), at two different time points (24 h and 48 h). These data suggest that there is no negative effect on the survival of BMSCs when seeding on PLGA-GO and suffering from PA treatment (10 mJ).

### PA promoted the osteogenic differentiation of BMSCs, without affecting their proliferation

**Figure 3:**
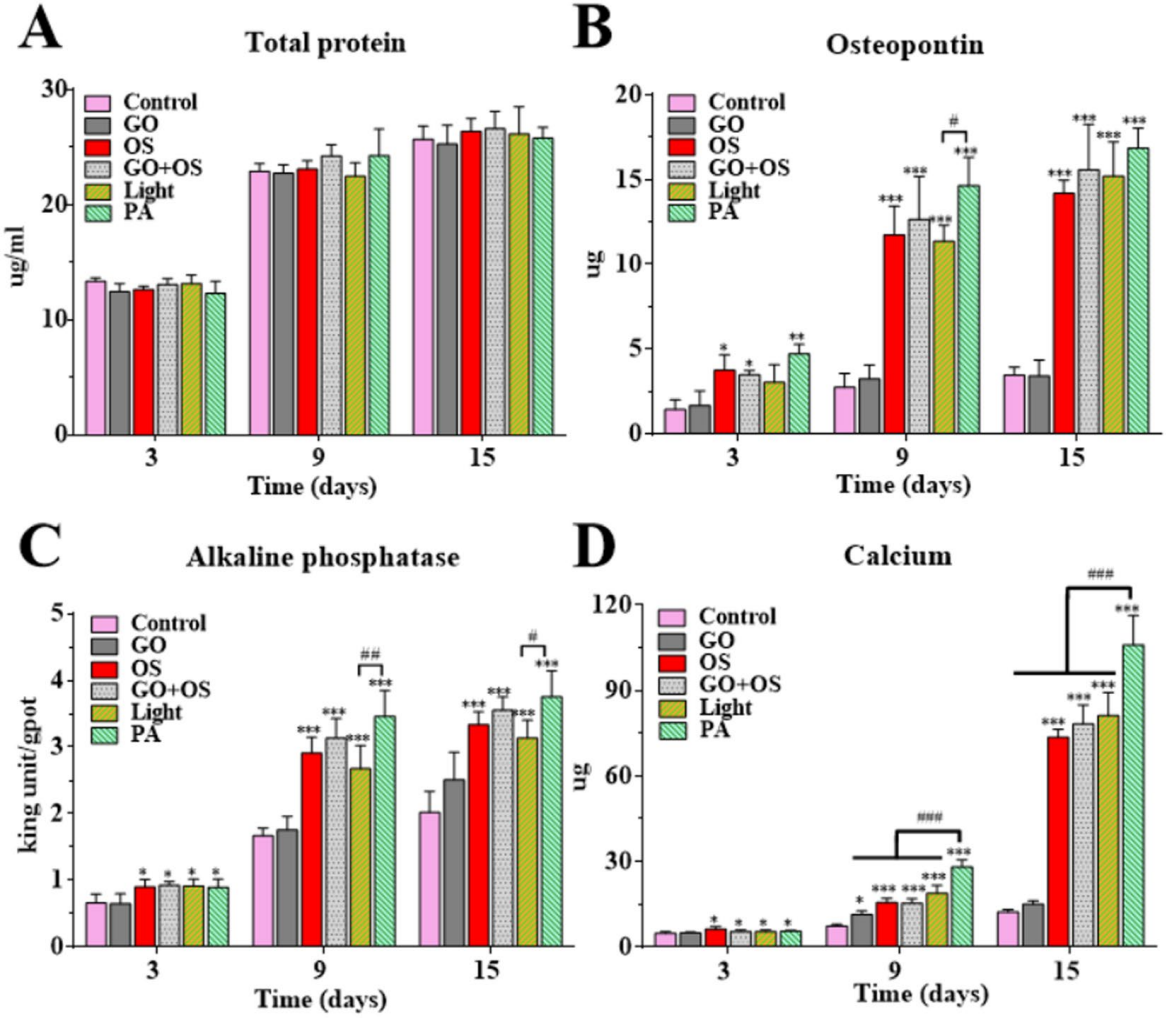
PA-pretreatment enhances the osteogenic differentiation of BMSCs *in vitro*. (**A**) The time course expression profiles of total protein in the indicated groups. The osteogenic markers of each experimental group, including osteopontin (**B**), alkaline phosphatase activity (**C**) and calcium content (**D**) were used to evaluate the osteogenic differentiation of BMSCs by quantitative analysis. All quantitative data are presented as mean ± S.D, n = 4; *represents statistical difference as compared with Control group; ^#^represents statistical difference as compared with PA group. *P < 0.05, **P < 0.01, ***P < 0.001; ^#^ *P* < 0.05, ^##^ *P* < 0.01; ^###^ *P* < 0.001 from One-way ANOVA with Student–Newman-Keuls *post hoc* test.

As mineralization and maturation of extracellular matrix depend on both the proliferation and differentiation of stem cells, the cell total protein was detected at 3, 9 and 15 days to monitor the growth status of cells. There was no significant difference in the total protein between groups at all time points (Fig. 3A). Besides, comparing of total proteins at different times, the growth peak of total proteins arises during the period between 3 days and 9 days. However, the total proteins of each group did not significant increase from day 9 to day 15 (Fig. 3A), possibly due to the limitation of growth space.

Osteopontin (OPN), an early maker indicating osteo-differentiation, was secreted into culture medium during the progress of osteo-differentiation and was deemed as the essential factor for matrix mineralization by promoting cells attachment. The PA group has a highest expression of OPN, as compared with other groups throughout the experiment (Fig. 3B). Moreover, the OS, GO + OS and Light groups stayed a high-level whereas Control and GO groups showing a low-level of Osteopontin secretion in culture medium at all the time points (Fig. 3B). To compare the Light and PA groups, there was significant difference between them at day 9 (Fig. 3B). We also noted that the expression of OPN in OS, GO + OS, Light and PA groups exhibited an increase, a plateau (day 3 to day 9), then a stagnation (day 9 to day 15).

As an important marker of osteo-differentiation in BMSCs, ALP activity reached its peak at the end of the proliferative stage and before matrix maturation^34^. Figure 3C shows the ALP activity in all the groups at 3, 9, and 15 days under different culture conditions. The ALP activity of PA, Light, GO + OS and OS groups (Table 1) were obviously higher than the Control group at all time points (Fig. 3C). Among laser stimulation groups, PA group showed significantly greater expression than Light group at 9 days and 15 days, respectively (Fig. 3C). Besides, in OS, GO + OS, Light and PA groups, the ALP activity peaks between 3 days and 9 days and the increase slope was reduced during 9 to 16 days, indicating that the osteo-differentiation was significantly enhanced at early time point.

**Table 1:** Grouping details for the *in vitro* experiments.

Group	OS	GO	Laser irradiate
Control	−	−	−
GO	−	+	−
OS	+	−	−
GO + OS	+	+	−
Light	−	−	+
PA	−	+	+

OS: Osteogenic induction medium. GO: PLGA−GO scaffold.

PA group also shows a significantly higher calcium content than other groups all of time points, particularly at 15 days post treatment (Fig. 3D). Besides, the Light, GO + OS and OS groups, showed apparently higher calcium deposition, as compared with the Control group at all time points (Fig. 3D). Therefore, the increased deposition of calcium was occured in the late stage (day 15) of the positive groups (PA, Light, GO + OS and OS), supporting the notion that the calcium mineralization is a late stage marker for osteo-differentiation^35^.

### Alizarin red stainings further support that the osteogenic differentiation of BMSCs seeding on PLGA-GO is enhanced by PA treatment

**Figure 4:**
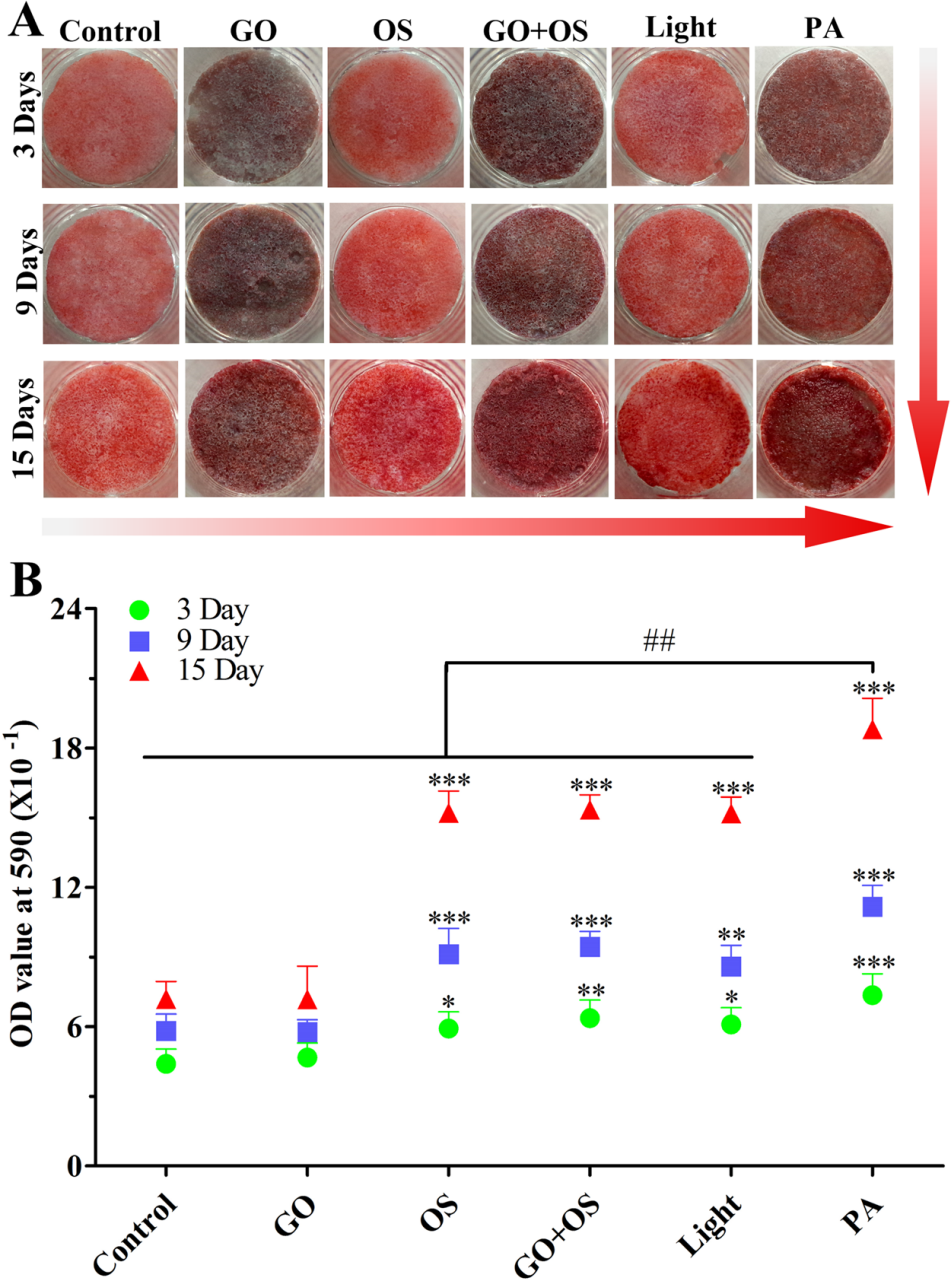
Alizarin red staining and its quantitative measurement further support that the osteogenic differentiation of BMSCs is enhanced by PA treatment. (**A**) The Alizarin red staining show calcium deposition in each experimental group (Control, GO, OS, GO + OS, Light, PA). (**B**) The mineralization of BMSCs were quantified by measuring the absorbance at 590 nm wavelength at 3, 9 and 15 days. All quantitative data were presented as mean ± SD, n = 4; *represents statistical difference as compared with Control group; ^#^represents statistical difference as compared with PA group at 15 days. *P < 0.05, **P < 0.01, ***P < 0.001; ^##^ *P* < 0.01 from One-way ANOVA with Student–Newman-Keuls *post hoc* test.

To further confirm the calcium deposition of each group, alizarin red staining was performed in all the groups (Fig. 4A). As compared with the Control group, we found that the PA, Light, GO + OS and OS groups, particularly the PA group, showed more mineralization of extracellular matrix over time. OS, GO + OS and Light groups were equivalent to each other in stain, whereas PA group showed more calcium matrix deposition at both 9 and 15 days. To quantitatively measure the calcium concentration, calcium matrix deposition of each well was dissolved by adding 1 mol/L HCl (200 µL) and detected by a microplate reader at 520 nm wavelength (Fig. 4B). These results indicate that the calcium matrix deposition of PA group distinctly preceded over the other groups, while all the OS, GO + OS and Light groups were significantly greater than Control group (Fig. 4A,B).

### PA-pretreated PLGA-GO seeded with BMSCs efficiently enhanced bone defect repair *in vivo*

**Figure 5:**
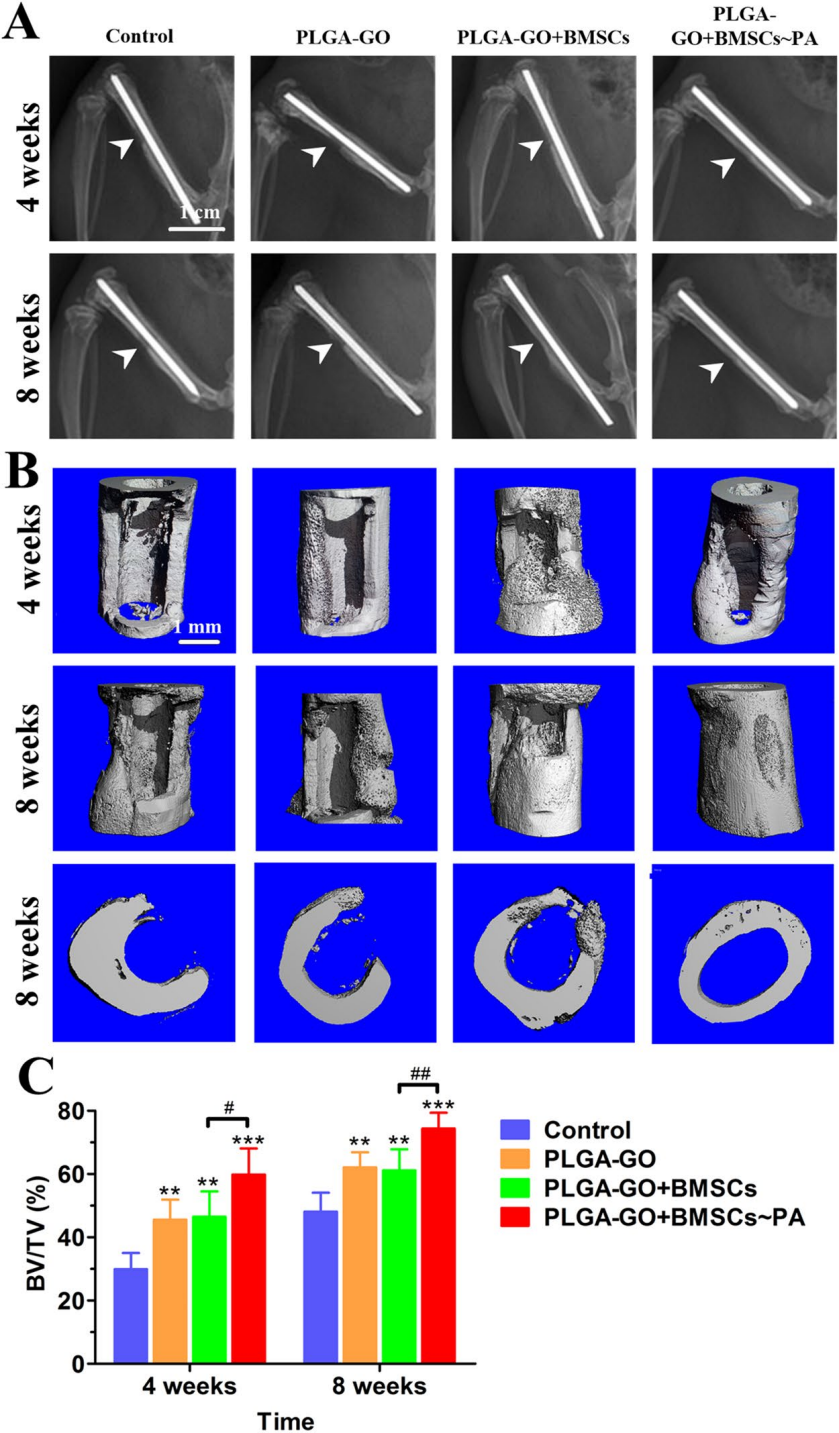
PA-pretreated PLGA-GO seeding with BMSCs significantly enhances the repair of bone defect *in vivo*. (**A**,**B**) Radiographs (**A**) and micro-CT 3D images (**B**) show new bone formation in bone defects of Control, PLGA-GO, PLGA-GO + BMSCs and PLGA-GO + BMSCs~PA groups at weeks 4 and 8 after implantation. The white arrows identify the bone defects. (**C**) BV/TV was quantified to analyze new bone formation within the bone defects. All quantitative data were presented as mean ± SD, n = 5; *represents statistical difference as compared with Control group; ^#^represents statistical difference as compared with PA group. *P < 0.05, **P < 0.01, ***P < 0.001; ^#^ *P* < 0.05, ^##^ *P* < 0.01 from One-way ANOVA with Student–Newman-Keuls *post hoc* test.

We applied a bone defect model created at the mid-shaft of femur of rat to further investigate the potential of the PA-pretreated PLGA-GO seeded with BMSCs in the regeneration of new bone tissue *in vivo* (Fig. 1C), as previously reported protocol^36^. Radiographs clearly demonstrated the regeneration of bone defect in each group at 4 and 8 weeks post-surgery (Fig. 5A). 3D micro-CT images showed the new bone formation within the defect site at 4 and 8 weeks after surgery (Fig. 5B). Among the scaffold implantation groups (Table 2), at week 4 post-surgery, mineralized bone tissue was appeared within the scaffold at the defect site; at week 8 after surgery, abundant mineralized bone was formed at the defect site. Particularly in the PLGA-GO + BMSCs~PA group, the defect region was filled with mineralized callus and bridged (Fig. 5B). However, the bone defect of control group did not heal, presenting with no significant bone formation at week 4, and only a few mineralized bones could be found on the edge of bone defect up to week 8 (Fig. 5C). These results indicate that the PLGA-GO scaffold can promote the healing of bone defect, and this effect would be further enhanced after combining with PA treatment.

**Table 2:** Grouping details for the *in vivo* experiments.

Group	PLGA-GO scaffold	BMSCs	Laser irradiate
Control	−	−	−
PLGA-GO	+	−	−
PLGA-GO+BMSCs	+	+	−
PLGA-GO+BMSCs~PA	+	+	+

### Histological outcome of bone defect repair was significantly improved by combining PA effect with stem cells

**Figure 6:**
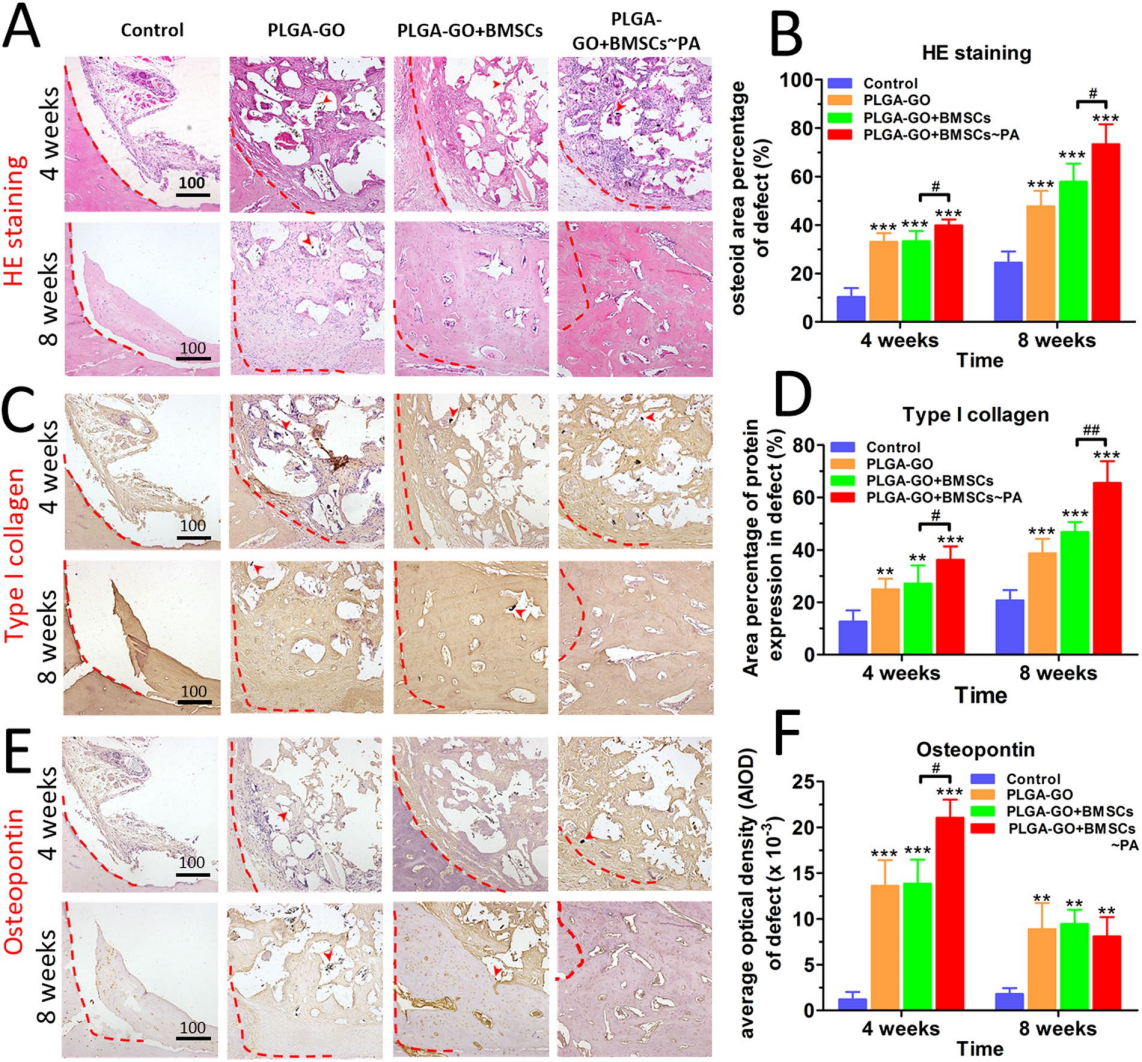
Histological and immunohistochemical results indicate best outcome in animals implanting with PA-pretreated PLGA-GO combined with BMSCs. H.E staining (**A**) and immunohistochemical staining of type I collagen (**C**) and OPN (**E**) for analyzing the new bone formation within the sites of bone defects. The red dashed line marked the edge of the bone defect while the red arrows pointed to the GO oxide particles. Panels B, D, F are quantitative data from panels A, C, E, respectively. All quantitative data were presented as mean ± S.D, n = 5; *represents statistical difference as compared with Control group at the same time point; ^#^represents statistical difference as compared with PA group at the same time point. *P < 0.05, **P < 0.01, ***P < 0.001; ^#^ *P* < 0.05, ^##^ *P* < 0.01 from One-way ANOVA with Student–Newman-Keuls *post hoc* test.

At week 4 post-surgery, in the scaffolds implanted groups, we found that both the osteoid, OPN and type I collagen have considerable expression on the scaffold but sloppy and formless (Fig. 6A–F). At week 8 post-surgery, significantly more new osteoid, type I collagen and OPN were found in the defect site with the scaffold degradation over time, as compared with the Control group (Fig. 6B,D,F). However, the control group presented with only a few immaturely callus generation around the edge of the bone defect site. Of note, the PLGA-GO + BMSCs~PA group showed much greater healing effect than the other groups at both 4 weeks and 8 weeks (Fig. 6A,C,E). These results indicate that PLGA-GO scaffolds indeed accelerated the healing of bone defect with or without the presence of BMSCs. However, bone regeneration was further promoted by the PA effect.

**Figure 7:**
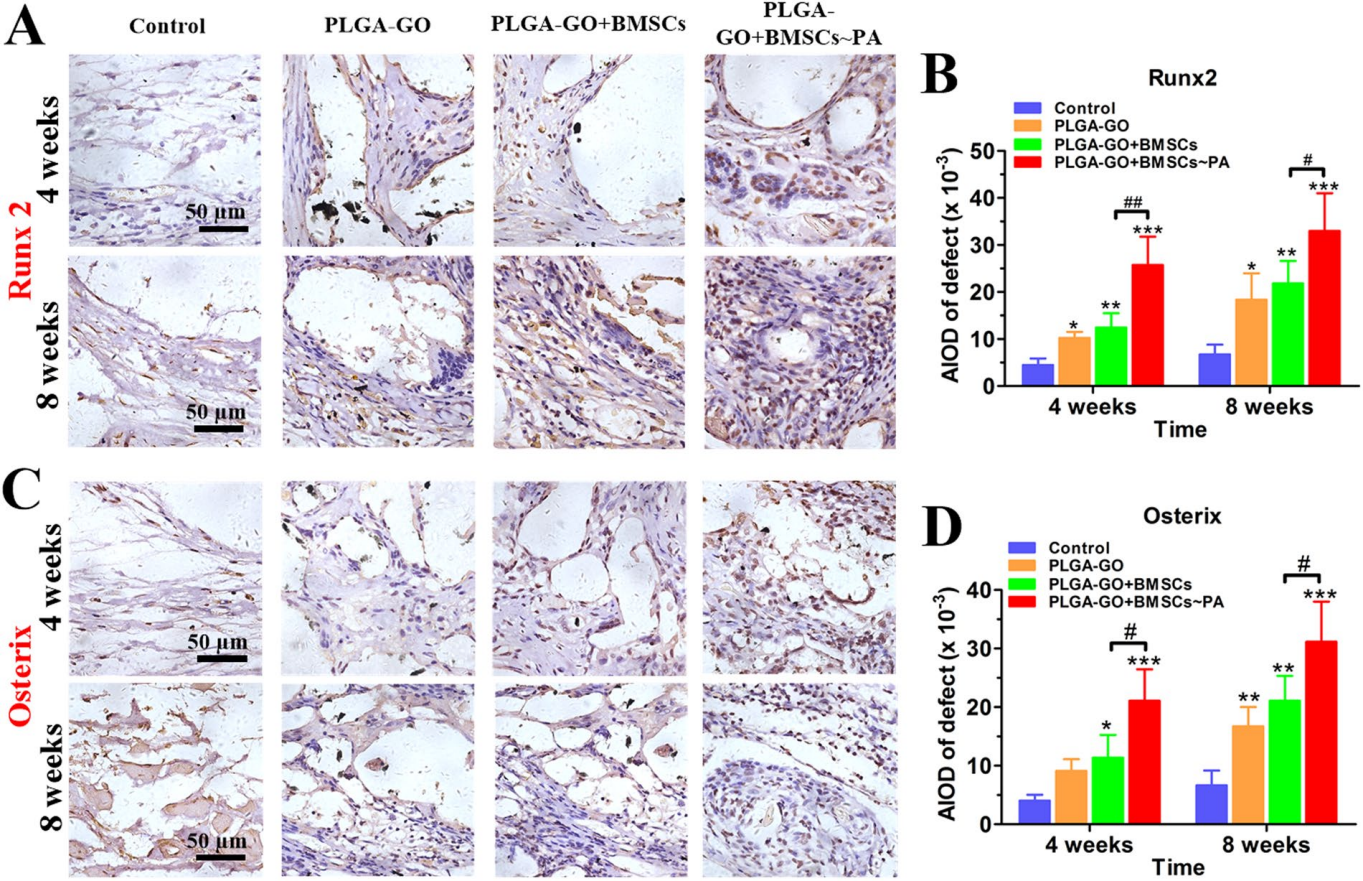
Both Runx2 and Osterix are significantly increased at the bone defect sites of animals implanting with PA-pretreated PLGA-GO combined with BMSCs. (**A**,**C**) Immunohistochemical staining of Runx2 and Osterix for estimating the new bone formation within the bone defects. The red dashed line marked the edge of the bone defect while the red arrows pointed to GO particles. Panels B and D were quantitative data from panels A and C, respectively. All quantitative data were presented as mean ± S.D, n = 5; *represents statistical difference as compared with Control group at the same time point; ^#^represents statistical difference as compared with PA group at the same time point. *P < 0.05, **P < 0.01, ***P < 0.001; ^#^ *P* < 0.05, ^##^ *P* < 0.01 from One-way ANOVA with Student–Newman-Keuls *post hoc* test.

Runx2 is the most important transcriptional factor controlling osteoblastic differentiation and binding to specific promoters and regulates transcription of numerous osteoblastic genes and up-regulates the expression of osteocalcin, osteopontin, and bone sialoprotein, which play a crucial role in bone repair. Osterix is a zinc finger-containing transcription factor acting as the downstream of Runx2, which regulates and promotes osteoblast maturation and stability, is also essential for osteoblast differentiation^37–39^. Both factors play a key role in the development and maturation of osteogenic differentiation^40^. Thus, we performed the immunohistochemistry to investigate the expression profiles of Runx2 and Osterix in bone defect. The PLGA-GO + BMSCs~PA group showed much significantly higher expression of Runx2 and Osterix, as compared to the other groups (Fig. 7A–D). Additionally, the scaffold implantation groups show higher expression than Control group (Fig. 7A–D). These results indicate that the PLGA-GO + BMSCs~PA group hold a strongest osteogenesis behavior and potentiality among groups and all scaffolds implanted groups showed significantly better outcome than the Control group.

### Neovascularization within the bone defect site was enhanced by PLGA-GO and PA effect

**Figure 8:**
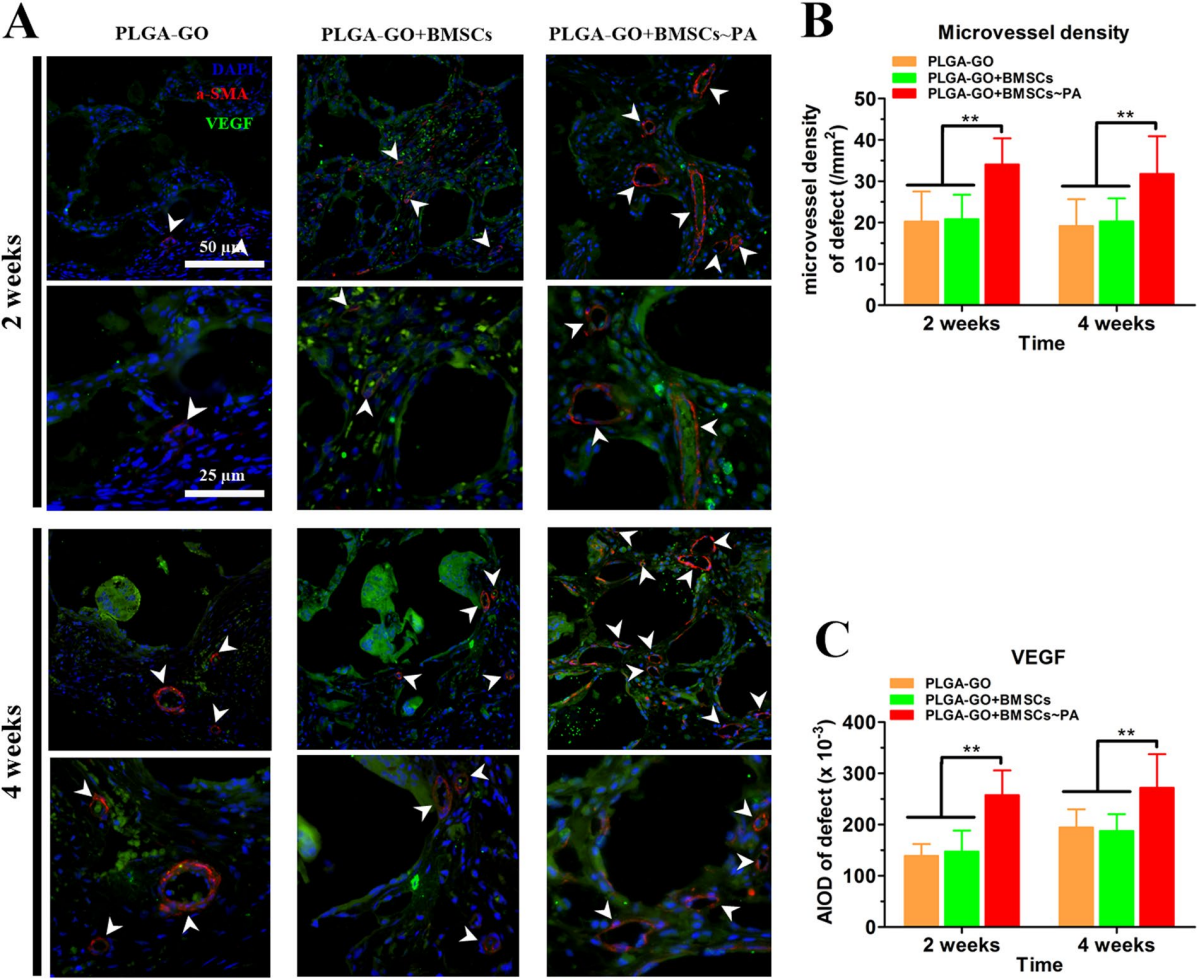
PA treatment significantly induces neovascularization within at the bone defect sites of animals implanting with PA-pretreated PLGA-GO combined with BMSCs. (**A**) Immunofluorescence assays show the α-SMA (Red color) and VEGF (Green color) expression in PLGA-GO, PLGA-GO + BMSCs and PLGA-GO + BMSCs~PA groups after implantation 2 weeks and 4 weeks. White arrows point to the α-SMA positive micro-vessels. (**B**) Mean microvessel density at the bone defect were quantified for statistical analysis. (**C**) The VEGF expression was quantified by average fluorescence intensity. All quantitative data were presented as mean ± S.D, n = 5; **P < 0.01, as compared with PA group at the same time point, from One-way ANOVA with Student–Newman-Keuls *post hoc* test.

Since bone is a highly vascularized organ and bone repair requires a large amount of blood supply and nutrition, angiogenesis plays an important role in osteogenesis^41^. Vascular endothelial growth factor (VEGF), one of the most important growth factors regulating the vascular development and angiogenesis, can enhance bone repair directly or indirectly by promoting angiogenesis and bone metabolism^41–43^. Therefore, the expression profiles of VEGF and α-SMA at 2 weeks and 4 weeks post-implantation were measured by the immunofluorescence assays to evaluate the neovascularization within the bone defect site. The neovascularization within the bone defect regions were demonstrated in Fig. 8A. More newly formed micro-vessels and stronger fluorescence of VEGF were observed in the defects of the PLGA-GO + BMSCs~PA group to compare with the other groups. New vessel ingrowth and VEGF were significantly increased in all groups from 2 to 4 weeks (Fig. 8A,B). Therefore, these results suggest that the PLGA-GO scaffold can elevate the VEGF secretion and angiogenesis and this effect could be greatly enlarged after incorporating PA effect with BMSCs.

## Discussion

In our *in vivo* study, after eliminating the potential effects of PA on cell proliferation in each group, based on the expression profiles of key osteogenic markers, including OPN, alkaline phosphatase and calcium deposition, we found that (**a**) the PA effect generated by the PLGA-GO scaffold can promote osteogenic differentiation of BMSCs; (**b**) pulsed laser stimulation (200 ns, 10 mJ and 10 Hz) without GO contribute to promote BMSCs osteogenic differentiation and this effect was further enhanced by incorporating with GO; (**c**) among all groups, the PA group has the best performance in the induction of osteogenic differentiation of BMSCs. However, as compared to the GO with Control group, PLGA-GO scaffolds did a poor performance in enhancing BMSCs osteogenic differentiation, which is inconsistent with some studies claiming that GO has a positive effect on the osteogenic differentiation of BMSCs^44^. We proposed that these distinct results were caused by that most of the GO in PLGA did not directly contact with BMSCs. Another possibility is for reducing the cytotoxicity of GO, we have used rather low concentration of GO in our study. In the *in vivo* study, analysis of bone defects by X-ray, micro-CT and immunohistochemistry, we find that PLGA-GO scaffolds paly a osteo-conductive role in bone regeneration and the PA treatment significantly enhance the repair of bone defect in rats through inducing osteogenic differentiation of primary cells as well as promoting the neovascularization within the site of bone defect. A number of studies have confirmed that BMSCs can promote bone formation under appropriate conditions. However, there was no significant difference between the PLGA-GO and PLGA-GO + BMSCs groups of bone defect healing in vivo study. Some reports declare that PLGA-GO scaffolds have the ability to promote cell adhesion and growth^12^. Moreover, the result shown in Fig. 2D in our study also demonstrated that BMSCs could adhere and grow well on PLGA-GO scaffolds. When the PLGA-GO scaffolds were implanted in bone defects in rats, BMSCs in the bone marrow cavity could sufficiently contact and adhere to the surface of PLGA-GO scaffolds. Therefore, we proposed that some endogenous BMSCs could adhere to and spread well on PLGA-GO scaffolds implanted in rats, which make the PLGA-GO and PLGA-GO + BMSCs groups have similar outcome in bone defect healing. Anyway, to get more information and overcome some limitations of this study, future study is needed.

Allografts or autografts, as the traditional remedies for bone defect, provide curative effect to the defect but often also cause some challenging problems. It has prompted us to explore a more effective treatment for the bone defect and nonunion following the development of tissue engineering. Based on the features which have multiple differentiation potentials with the guidance of appropriate micro-environment^45^, it’s reasonable to choose BMSCs as the seeding cells to cure bone defect and nonunion. The PLGA-GO scaffold not only provides with the superior aperture structure, but also acts as an ideal material mediating the PA effect. Moreover, Nano-graphene oxide provides good dispersion in polymer matrixes and enhances the structural stability and biocompatibility of scaffold^12^. Good plasticity, physicochemical and biological properties enable it to be processed into various shapes to fill the specific size of bone defect. On the other hand, as a physical stimulation, different from drugs and biologically active ingredients, the PA effect is expected to impact on BMSCs more stable and balanced, without remnant and metabolic products. Therefore, we have fabricated a special biological composite porous scaffold as a carrier for BMSCs incorporated with PA effect to improve bone defect healing.

In clinical application, the PLGA has been widely used in tissue engineering for its good biocompatibility and structural performance^46^. These advantages have been further expanded by combining with GO. PLGA-GO scaffolds could be controlled by changing the pore structure of PLGA and the content of GO for adapting to different tissue defects and therapeutic purposes. In addition, PA treatment has been applied to the tissue engineering by its pertinence, efficiency and penetration, previously studies demonstrate that PA can promote cell proliferation and differentiation or killing tumour cell by regulating the power of laser^20,47^. Recently, there are some reports that diagnosis and treatment of tumors by PA therapy for its imaging characteristics and photothermal effect^48,49^. Thus it is of a great potential to apply as an innovative approach by incorporating biological materials and factors. Our study may put forward a new idea for exploring more effective treatment of bone defect by making good use of PA effect, though further studies will be carried out in the coming future.

## Materials and Methods

### Materials

Graphite and thionyl chloride (SOCl_2_) were purchased from Aladdin (Shanghai, China) and Xiya (Shandong, China), respectively. Hydroxyl-terminated Poly (L-Lactide-Co-Glycolide) (PLGA, MW = 150,000, L-lactide: glycolic acid = 9:1) was supplied by Daigang (Shandong, China).

### Fabrication of scaffolds

Graphite oxide was prepared by oxidation of graphite powder via an improved Hummers’ method^50^. H_2_SO_4_/H_3_PO_4_ (140 mL, v/v, 9:1) was added to a mixture of graphite powder (3 g) and KMnO_4_ (6 g) with vigorous stirring. The mixture was heated to 50 ^°^C and stirred for 12 h. After being cooled to room temperature, the reaction mixture was poured to distilled water (100 mL) in an ice bath, followed by addition of H_2_O_2_ (30%, 5 mL). The resultant suspension was centrifuged at 8000 rpm for 30 min and the precipitate was collected and repeatedly washed with water (100 mL), HCl (30%, 100 mL) and ethanol (200 mL). The solid was collected and dried in vaccum at room temperature for 12 h to get graphite oxide. Graphite oxide dispersion in DMF (3.2 mg/mL, 1 mL) was mixed with SOCl_2_ (10 mL) and refluxed for 12 h to convert the carboxylic acids to acyl chlorides^51^. After the removal of SOCl_2_ in graphite oxide dispersion, PLGA solution in DMF (0.2 g/mL, 10 mL) was added. After GO and PLGA were homogeneously mixed, DMF in the mixture was removed on a rotavapor. The PLGA-GO composites was dried in vacuum at 95 °C to remove the residual DMF.

PLGA-GO composite (2 g) was dissolved in CHCl_3_ (20 mL) and sieved NaCl (18 g, 200–300 μm in size) was added under vigorous stirring. When PLGA-GO and sieved NaCl seem to be homogeneous, CHCl_3_ was removed. The resultant solid was filled in a self-designed stainless steel mold and compressed under a static pressure of 10 MPa to yield a composite block. The composite block in mold was heated to 175 °C in an oven for 5 min^52^, followed by demolding at room temperature. The NaCl particles were removed from the PLGA-GO block by dialysis against distilled water for 3 days. PLGA-GO scaffolds were obtained upon drying the above block in air.

### Characterization on the biological properties of PLGA-GO scaffolds

The pore structure of PLGA and PLGA-GO scaffolds were studied under scanning electron microscope (SEM, JSM-6360LA, Japan). And the functional groups of PLGA and PLGA-GO were characterized by LabRam HR800 Raman spectroscopy (532 nm). The true density of the PLGA and PLGA-GO scaffolds was measured by True Density Analyzer (TD-2200), then the porosity of scaffolds could be calculated. In addition, the photoacoustic signal was obtained by a PA data acquisition system.

To evaluate cytocompatibility of PLGA-GO scaffold, BMSCs were seeded on the scaffold with 1.0 × 10^6^ cells per scaffold. After 72 h culturing in an incubator, the scaffolds and cells complexes were rinsed three times with PBS, fixed in 1.25% glutaraldehyde, followed by sequential dehydration in graded ethanol and were observed on the scaffolds by using the SEM finally^53^. Furthermore, to investigate the cytotoxicity of PLGA-GO scaffold with PA stimulation, we respectively seeded the BMSCs with an initially of 20,000 cells/well on PLGA-GO sheet culturing in 24-well tissue culture plates and divided them into two groups, that is, with or without the laser irradiation. One group was suffered 10 minutes of photoacoustic stimulation every 24 hours, while the other group cultivate in the same condition except PA stimulation. The CCK8 test (Dojindo, Japan) was performed to investigate the cell proliferation at 24 h and 48 h.

### Establishment of PA system

According to our and others’ previous studies, the optimal intensity and parameters for pulse laser were decided to 532 nm short pulsed laser with a pulse duration of 200 ns and a pulse energy of 10 mJ at a repetition rate of 10 Hz^17,20^. The horizontally 10 mJ pulsed laser beam irradiated on the PLGA-GO scaffolds after refracting by the three-prism. Then the PLGA-GO scaffolds absorbing the laser energy could produce physical sound waves which eventually affected the behaviors of the seeded BMSCs (Fig. 1B).

### *In vitro* treatments

The BMSCs were seeded initially at 20,000 cells/well on PLGA sheet or PLGA-GO sheet culturing in 24-well tissue culture plates with or without the osteogenic supplements and laser irradiation. According to the protocol of experiment, six experimental groups were design as the Table 1 shown that PLGA sheet without osteogenic supplements and laser stimulate (Control), PLGA-GO sheet without osteogenic supplements and laser irradiate (GO), PLGA sheet cultivating with osteogenic supplements without laser irradiate (OS), PLGA-GO sheet cultivating with osteogenic supplements without laser irradiate (GO + OS), PLGA sheet incorporation with laser irradiate without osteogenic supplements (Light) and PLGA-GO sheet incorporation with laser irradiate without osteogenic supplements (PA). The PA and Light groups were irradiated for 10 minutes a day. After 3, 9 or 15 days of repeated exposure, each group was surveyed the relevant indicators of osteogenic differentiation to evaluate the effect of PLGA-GO scaffold incorporated with PA stimulation for enhancing BMSCs osteogenic differentiation.

### Animal model and grouping

All experimental animals were supplied from the laboratory animal center of the Shantou University Medical College. The experimental protocol of this study was approved by the Animal Experimentation Ethics Committee of the Shantou University Medical College and all the animal handling and surgical procedures strictly abided by the rules and regulations of the animal care and use committee. And all experiment operations were performed with a proficient and temperate surgical plan.

Firstly, the scaffold was immersed in the cell suspension with a concentration of 1.0 × 10^6^ cells/ml for 4 h. Then the scaffold and cell complexes were removed into culture dish cultivating with or without PA stimulation for 9 days *in vitro* (PLGA-GO + BMSCs~PA and PLGA-GO + BMSCs groups). And some of PLGA-GO scaffolds were immersed in the medium for 9 days without BMSCs and PA stimulation (PLGA-GO group) before transplanted into animal models.

In our *in vivo* experiments, a total of 40 rats were used. we adopted a segmental femoral bone defect model in adult male Sprague Dawley rat^36,54^. A critical sized defect of left femoral bone midshaft with a length of 5 mm and one half of the circled cortex of the femur was surgically removed for scaffold implantation (Fig. 1C). Briefly, 4-month-old male SD rats with the weight between 350–400 g were chosen as subject and were randomly divided into control, PLGA-GO scaffold (PLGA-GO), PLGA-GO scaffold with the BMSCs (PLGA-GO + BMSCs) and PLGA-GO scaffold with the BMSCs incorporation the processing of PA stimulation (PLGA-GO + BMSCs~PA) (Table 2). By intraperitoneal injection of sodium pentobarbital (40 mg/kg body weight) for general anaesthesia, a 2-3 cm incision was made to separate and expose the femur after skin preparation and disinfection. Then the specific size defect of the femoral bone midshaft was removed while the PLGA-GO scaffolds with an applicable size was implanted. The surgery spot was stratified sutured finally.

All animals were kept at 20–25 °C with a constant humidity and allowed food and water *ad libitum*. After 2, 4, 8 weeks, the samples of each group were collected to evaluate bone formation by using X-ray, Miro-CT and Immunohistochemistry.

### Primary cell isolation and culture

The BMSCs were obtained from 3-month-old SD rat by separating the femoral bone marrow cells^13^. Briefly, we took their tibias and femurs to flush the bone marrow in the cavity with the culture medium (Gibco) supplemented with 10% fetal bovine serum (FBS, Gibco), 1% penicillin/streptomycin (Gibco) in an aseptic condition after the rat were euthanized. The cell suspension was cultivated in a 10 cm diameter petri dishes and put into an incubator at 37 °C with 5% CO_2_. After 72 h attaching, the suspended cells were removed by rinsing with phosphate buffer saline (FBS), and the adhered cells were cultured and passaged with a rate of 1:3 when Cell density reach around 90%. We used BMSCs at passages 3–5 for the following experiments.

### Alkaline phosphatase assay

To quantify the alkaline phosphatase activity, ALP assay (PanEra AAPR279) was performed at 3, 9 and 15 days. Briefly, 30 µL cell lysates of each sample or standard solution was added to 96-well plate in triplicate, then 100 µL reaction buffer solution was added. For incubating 15 minutes at 37 °C, the plate was added into 150 µL chromogenic reaction liquid. Immediately, the absorbance of plate was read with microplate reader at 405 nm. According to the standard curve, the ALP activity of cells was counted finally.

### Osteopontin assay

Culture medium was collected and kept in the refrigerator of −80 °C for subsequent testing. Briefly, 50 µL of assay diluent and 50 µL of standard or samples were added to 96 wells that were percolated with an OPN polyclonal antibody allowing the OPN to bind and incubated for 2 h at room temperature. Then the samples and standards were aspirated, and 100 uL of an enzyme-linked polyclonal antibody reagent was added to each well an incubated at room temperature for 2 h and followed by aspiration; 100 µL of substrate solution was added to the wells for 30 minutes in the dark, causing a blue color when an enzyme reaction occurred, and the reaction was stopped with 100 µL of hydrochloric acid turning the samples yellow. Absorbance was determined at 450 nm on a microplate reader, and OPN level was determined by the comparison to the OPN standards.

### Calcium content assay

Calcium quantification was performed by adding 1 mol/L HCl (1 mL) to each sample and put on a shaker overnight for dissolving the calcium thoroughly. For the assay (PanEra AAPR313-1), using calcium chloride as the standard, 20 µL of samples or standard was added in triplicate into each well of a 96 well plate and 280 µL arsenazo III calcium assay reagent was then added. Absorbance was determined at 590 nm on a microplate reader.

### X-ray and CT evaluation of new bone formation

To monitor the progress of bone defect repair, the rats were taken X-rays every week at post-operation. At 2, 4 and 8 weeks, the rats were sacrificed by cervical dislocation. Then their left femora were collected and fixed in 10% formalin 24 h at room temperature and scanned by micro-CT to observe and estimate new bone formation within the site of bone defect.

### Histological evaluation of new bone formation

Concurrently, histochemical and immunohistochemical analysis were employed to verify the changes of local bone defect based on the X-rays and micro-CT. After X-ray and Miro-CT imaging, all the middle segments of femoral samples were decalcified in 10% ethylene diaminetetraacetic acid (EDTA) for 6 weeks, changed the solution of EDTA every 2-3 days, followed dehydrated in a series of ethanol. The orientation and alignment of femurs were carefully made appropriate adjustment to insure a significative view of the defect during paraffin embedding. Longitudinal serial sections, 4 µm in thickness, were cut and mounted on microscope slides. For general histological studies, hematoxylin and eosin (H&E) staining, type I collagen kit (Col I) and OPN were performed per our previous protocol^36^. As osteogenic transcriptional factor, Runx2 and Osterix protein expression levels were also detected by immunohistochemistry. Furthermore, α-SMA and VEGF were quantified by immunofluorescence to estimate the neovascularization within the bone defect site.

### Statistical analysis

Referring to relevant studies^55,56^, we selected the micro-CT test as a representative and used PASS 11.01 software to calculate Power. A one-way ANOVA design with 3 treatment groups and one control group has an average group sample size of 5 for a total sample size of 20, in which we selected CT outcome as the representative variable. Both the 4 weeks group and 8 weeks group design achieved an any-pair power of 0.885 and an all-pair power of 0.944 using the Dunnett’s Test procedure for comparing each treatment mean with the control mean. All quantitative data are presented as mean ± S.D. Setting significant difference at *P* < 0.05, statistical differences were analyzed by one-way ANOVA with indicated *post hoc* tests by using GraphPad Prism software (Version 6.01).

## Electronic supplementary material

Supplementary information
