# Supplementary material for: Photoacoustic stimulation promotes the osteogenic differentiation of bone mesenchymal stem cells to enhance the repair of bone defect
Source: Sci Rep. 2017 Nov 20;7:15842. doi: 10.1038/s41598-017-15879-4 (PMC5696557; doi:10.1038/s41598-017-15879-4)
Supplement: Supplementary file 1 — Supplementary information [file 41598_2017_15879_MOESM1_ESM.pdf]

Supplementary data for

Photoacoustic stimulation promotes the osteogenic differentiation of bone mesenchymal stem cells to enhance the repair of bone defect

Zebin Huang[a, 1], Jiankun Xu[a, b, 1], Jiebin Chen[a, 1], Hongjiang Chen[a], Hailong Wang[c], Zhonglian Huang[a], Youbin Chen[a], Xiaolin Lu[a], Fushen Lu[c, *] & Jun Hu[a, **]

[a] Department of Orthopaedics, the First Affiliated Hospital, Shantou University Medical College, Guangdong Province, China.

[b] Department of Orthopaedics and Traumatology, Prince of Wales Hospital, Faculty of Medicine, the Chinese University of Hong Kong, Hong Kong SAR, China.

[c] Department of Chemistry and Key Laboratory for Preparation and Application of Ordered Structural Materials of Guangdong Province, Shantou University, Guangdong, China.

[1] These authors contributed equally to this work.

Correspondence and requests for materials should be addressed to FS. L. (email: fslu@stu.edu.cn)* or J.H. (email: hjzkm@vip.163.com)**

Supplementary Figure 1

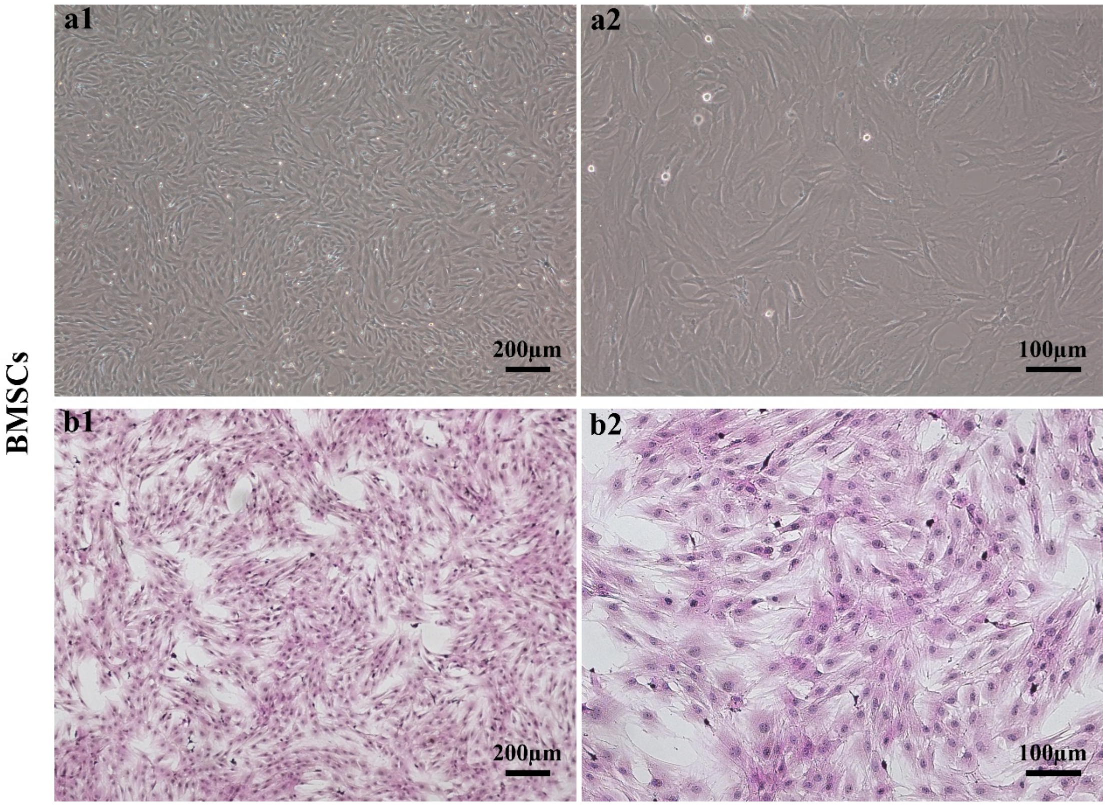

Supplementary Figure 1. (a) The cell morphology of bone marrow mesenchymal stem cells at passage 3 was observed by optical microscope. (b) H.E staining for the bone marrow mesenchymal stem cells at passage 3.

Supplementary Figure 2

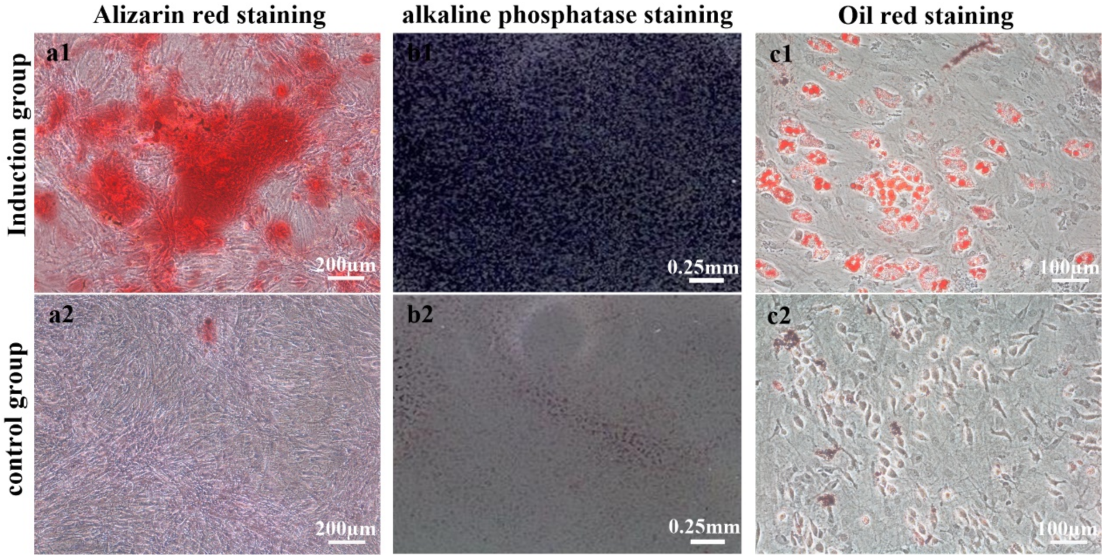

Supplementary Figure 2. We used corresponding induction medium to induce the BMSCs differentiate into osteoblasts and adipocytes respectively, indicating that the isolated cells were stem cells. (a1), (b1) and (c1) are representative images of the alizarin red staining, alkaline phosphatase staining and Oil red staining, respectively, while the (a1), (b1) and (c1) are the control group without induction medium.

Supplementary Figure 3

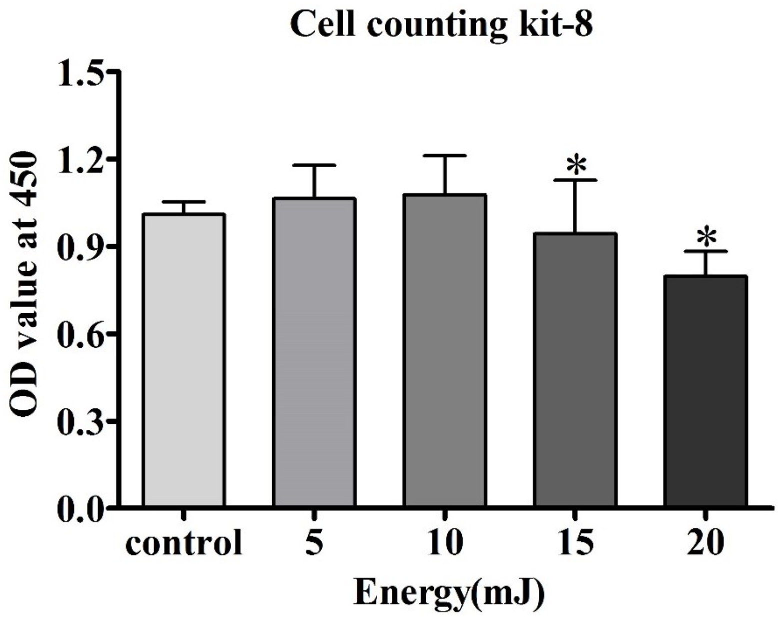

Supplementary Figure 3. The effects of different intensities on the proliferation of BMSCs. Setting up the 5 mJ, 10 mJ, 15 mJ, 20 mJ and control groups, the 10 mJ group showed similar OD value as the control group, indicating that the cell vitality was not affected under the treatment of this energy. All data represented the mean ± SD, n = 3; * represents statistical difference as compared with control group by One-way ANOVA (*P < 0.05).

Supplementary Figure 4

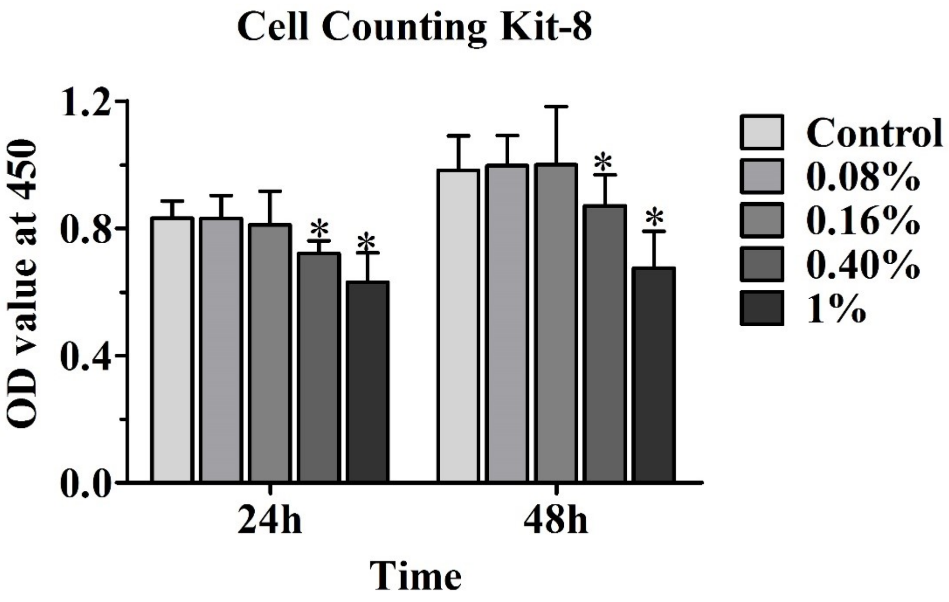

Supplementary Figure 4. 0.08% and 0.16% GO in PLGA groups showed the equivalent OD value as the control group. Higher than 0.4% GO leads to decline in cell viability. All data represented the mean ± SD, n=3; * represents statistical difference as compared with control by One-way ANOVA (*$P < 0.05$).
